# Artificial intelligence analysis of the single-lead ECG predicts long-term clinical outcomes

**DOI:** 10.1093/ehjdh/ztaf057

**Published:** 2025-06-09

**Authors:** Abdullah Alrumayh, Patrik Bächtiger, Arunashis Sau, Josephine Mansell, Melanie T Almonte, Karanjot Chhatwal, Fu Siong Ng, Mihir A Kelshiker, Nicholas S Peters

**Affiliations:** National Heart and Lung Institute, Imperial College London, Du Cane Road, London W12 0HS, UK; Department of Cardiology, Imperial College Healthcare NHS Trust, Du Cane Road, London W12 0HS, UK; Department of Basic Sciences, Prince Sultan Bin Abdulaziz College for Emergency Medical Services, King Saud University, Riyadh, Saudi Arabia; National Heart and Lung Institute, Imperial College London, Du Cane Road, London W12 0HS, UK; Department of Cardiology, Imperial College Healthcare NHS Trust, Du Cane Road, London W12 0HS, UK; National Heart and Lung Institute, Imperial College London, Du Cane Road, London W12 0HS, UK; Department of Cardiology, Imperial College Healthcare NHS Trust, Du Cane Road, London W12 0HS, UK; National Heart and Lung Institute, Imperial College London, Du Cane Road, London W12 0HS, UK; Department of Cardiology, Imperial College Healthcare NHS Trust, Du Cane Road, London W12 0HS, UK; National Heart and Lung Institute, Imperial College London, Du Cane Road, London W12 0HS, UK; Department of Cardiology, Imperial College Healthcare NHS Trust, Du Cane Road, London W12 0HS, UK; Department of Epidemiology and Biostatistics, School of Public Health, Imperial College London, London, UK; National Heart and Lung Institute, Imperial College London, Du Cane Road, London W12 0HS, UK; National Heart and Lung Institute, Imperial College London, Du Cane Road, London W12 0HS, UK; Department of Cardiology, Imperial College Healthcare NHS Trust, Du Cane Road, London W12 0HS, UK; National Heart and Lung Institute, Imperial College London, Du Cane Road, London W12 0HS, UK; Department of Cardiology, Imperial College Healthcare NHS Trust, Du Cane Road, London W12 0HS, UK; National Heart and Lung Institute, Imperial College London, Du Cane Road, London W12 0HS, UK; Department of Cardiology, Imperial College Healthcare NHS Trust, Du Cane Road, London W12 0HS, UK

**Keywords:** Cardiology, Artificial Intelligence, Digital Health, Public Health

## Abstract

**Aims:**

Artificial intelligence (AI) applied to a single-lead electrocardiogram (AI-ECG) can detect impaired left ventricular systolic dysfunction [LVSD: left ventricular ejection fraction (LVEF) ≤ 40%]. This study aimed to determine if AI-ECG can also predict the two-year risk of major adverse cardiovascular events (MACE) and all-cause mortality independent of LVSD.

**Methods and results:**

Clinical outcomes after two-year follow-up were collected on patients who attended for routine echocardiography and received simultaneous single-lead-ECG recording for AI-ECG analysis. MACE and all-cause mortality were compared by Cox regression, measured against the classification of LVEF > or ≤40%. A subgroup analysis was performed on patients with echocardiographic LVEF ≥ 50%. With previously established thresholds, ‘positive’ AI-ECG was defined as an LVEF-predicted ≤40%, and negative AI-ECG signified an LVEF-predicted >40%; 1007 patients were included for analysis (mean age, 62.3 years; 52.4% male). 339 (33.7%) had an AI-ECG-predicted LVEF ≤ 40% and had a higher MACE rate (LVEF ≤ 40% vs. >40%: 34.2% vs.11.9%; adjusted hazard ratio (aHR) 1.93; 95% CI, 1.39–2.69; *P* < 0.001), primarily driven by increased mortality (23% vs. 9.6%; *P* < 0.001; aHR 1.56; 95% CI, 1.06–2.29; *P* = 0.0239). In patients with echocardiographic LVEF ≥ 50%, there was a higher incidence of MACE in those with an AI-ECG ‘false positive’ prediction of LVEF ≤ 40% (27.2% vs.11.9%; *P* < 0.001; aHR 1.71 and 95% CI, 1.11–2.47) and all-cause mortality (20.4% vs. 9.6%; *P* < 0.001; aHR 1.59, 95% CI, 1.09–2.42).

**Conclusion:**

An AI-ECG algorithm designed to detect LVEF ≤ 40% can also identify patients at risk of MACE and all-cause mortality from single-lead ECG recording—independent of actual LVEF on echo. This requires further evaluation as a point-of-care risk stratification tool.

## Graphical Abstract

**Graphical Abstract:**
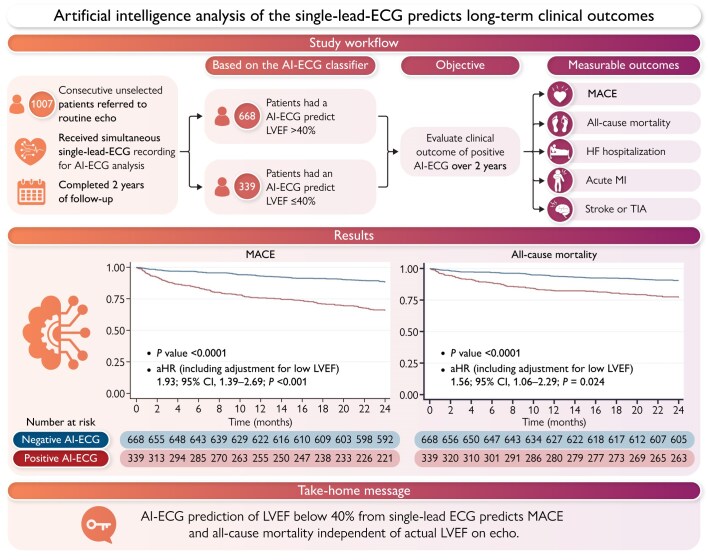

## Introduction

Recent advancements in artificial intelligence (AI) have led to the development of tools capable of accurately identifying various cardiac conditions.^1–6^ Among the most established use cases, analysis of the electrocardiogram (ECG) using AI algorithms (henceforth AI-ECG) applied to both 12-lead ECG and single-lead ECG can effectively identify individuals with an LVEF of 40% or lower—indicating left ventricular systolic dysfunction (LVSD).^1,3^ AI algorithms applied on a 12-lead ECG have also been found to correlate with major adverse cardiovascular events (MACE) and all-cause mortality independent of obvious confounders.^7,8^ In previous work, we completed an external validation study to measure the performance characteristics of AI-ECG to detect LVEF ≤ 40% from single-lead ECG alone.^3^ Sustained performance of AI-ECG with single- vs. 12-lead ECG underpins clinical application that would be scalable by virtue of low cost and simplicity; enabling access to novel risk prediction tools that could mitigate poor cardiovascular outcomes. Here, we test the hypothesis that this single-lead AI-ECG designed for the detection of LVEF ≤ 40% can accurately identify individuals at risk of MACE and all-cause mortality in patients with preserved LVEF. To further augment this, we also hypothesized that changes in a probability score as a continuous variable of the AI-ECG would be independently associated with increased risk of MACE and all-cause mortality.

## Methods

### Study design and participants

The DUO-EF-19 prospective study (NCT04601415) has been previously described.^3^ In brief, all adults (aged ≥18 years) across seven National Health Service (NHS) sites in London, UK, between February 2021 and May 2021 attending for routine transthoracic echocardiogram (echo) were eligible to participate. To summarize, in the DUO-EF 19 study (NCT04601415), single-lead ECGs were recorded from the precordium at well-established sites for cardiac auscultation (aortic, pulmonary, tricuspid, and mitral positions). The pulmonary valve position, most closely resembling the vector for Lead II of the conventional 12-lead ECG, provided the best detection performance and has henceforth been adopted as the standard precordial position for single-lead ECG recording for AI-ECG, including for this study. These patients had precordial single-lead ECG recordings at the time of echo. These were analysed by AI-ECG to classify the presence/absence of LVSD. For this study, we completed two years of follow-up through manual chart review for 1007 patients. The study included a single echo scan and a contemporaneous single ECG recording per patient (pulmonary position). This study was approved by the UK Health Research Authority (reference 21/LO/0051).

### AI-ECG algorithm

The AI-ECG algorithm has been previously described.^1,3,9^ In brief, the model utilizes a convolutional neural network that underwent training using a dataset consisting of 35 970 distinct pairs of 12-lead ECGs and echos. The model was originally developed at the Mayo Clinic and was trained on individual leads extracted from the 12-lead ECGs—the AI-ECG model architecture for ECG waveform processing used in this study has been extensively detailed in other research.^1,3^ The model was subsequently licenced to Eko Health (Emeryville, USA), who further modified it to work off single-lead ECG and accept phonocardiogram (PCG) as a supplementary input. Our study used Eko Health’s model which primarily uses single-lead ECG as input, but when available and of adequate quality, PCG serves as a secondary input. ECG recordings are classified as either adequate or poor quality for AI-ECG interpretation using a separate deep learning classifier trained on signal quality annotations.^3^ Any single-lead ECG determined to be of adequate quality will proceed to AI-ECG interpretation, regardless of the quality of secondary PCG input. The ECG-enabled stethoscope captures 15 s of single-lead ECG at a sampling rate of 500 Hz. Before analysis, the following filters are applied to the raw ECG signal—a notch filter to remove power line noise interference at 50/60 Hz from a mains supply, a high-pass filter (with cut-off frequency of 0.5 Hz) for baseline wander removal, and a Savitzky–Golay filter for smoothing. The AI-ECG's output ranges from 0 to 1, with 0.341 serving as the predetermined threshold (achieving prespecified sensitivity and specificity of >81 and >67, respectively) established in a previously published external validation study—Supplementary material online, Appendix  *Figure S1*.^3^ If the AI-ECG's output equals or exceeds 0.341, it signifies an LVEF ≤ 40%, denoting a ‘positive’ AI-ECG result—referred to as an AI-ECG classification output. The significance of an LVEF ≤ 40% lies in its indication of heart failure with reduced ejection fraction (HFrEF), which prompts the initiation of guideline-directed medical therapies. The AI-ECG value is also used for building a model ‘AI-ECG probability score’ (values ranging from 0 to 1) for predicting patients at risk of MACE and all-cause mortality. Eko Health had no role in the design, conduct, analysis, or presentation of the research detailed in this study. In order to determine whether the PCG input significantly affected the predictive ability, we compared the performance of AI-ECG using ECG + PCG input (the current method within the manufacturer's specification) against ECG input alone. All analyses presented were conducted by the authors.

### Outcomes

The primary outcome of the study was the occurrence of MACE during follow-up. MACE was defined as a combination of events, including all-cause mortality, acute myocardial infarction (MI), hospitalization due to HF, stroke, or transient ischaemic attack (TIA), during a follow-up period of up to 2 years. Additional analyses were conducted focusing on all-cause mortality as a standalone outcome and modified MACE, defined as a combination of all-cause mortality or hospitalization due to HF. The endpoint was evaluated for patients who had positive AI-ECG screens and those with negative AI-ECG screens in the entire cohort, regardless of their LVEF%.

Subgroup analyses were conducted to validate the findings specifically in patients with an echocardiographic LVEF of ≥50%. This was done to determine whether, in patients with an LVEF of ≥50%, an AI-ECG-predicted LVEF of ≤40% (positive AI-ECG) is predictive of MACE, all-cause mortality, and modified MACE. Cox regression analyses were conducted to evaluate AI-ECG results (both as a classification and as a probability score output) ability to predict MACE, all-cause mortality, and modified MACE risks.

### Statistical analysis

Demographic and clinical variables were summarized for the overall cohort using mean and standard deviations. We compared groups stratified by AI-ECG (positive vs. negative) using Student's *t*-tests for continuous variables or Pearson's χ^2^ test for categorical variables, as appropriate, with *P* < 0.05 considered statistically significant. Survival curves analysis was performed using Kaplan Meier estimate for patients with positive AI-ECG and negative AI-ECG with *P*-value reported from Log-rank test. Intercorrelation between variables was tested using Pearson or Spearman correlation, as appropriate. Univariate and multiple Cox proportional hazards models were used to account for variations in baseline characteristics. Confounding variables were selected using Akaike Information Criterion (AIC) bidirectional stepwise regression, with age and sex included in the model regardless of their contribution to AIC. The following variables were included in the Cox regression model, age, history of HF, LVEF%, AI-ECG (either as a classification [positive/negative] or as a probability score [0–1]), history of hypertension, MI, atrial fibrillation (informed by patient medical records), valvular heart disease, pacemaker, or cardiac intervention (e.g. coronary artery bypass graft surgery/percutaneous coronary intervention or valve surgery), diabetes mellitus, and chronic kidney disease. All these variables were collected based on a confirmed diagnosis on patient medical records; an echo indication alone was not considered sufficient to confirm of diagnosis. Hazard ratios (HR) with accompanying 95% confidence intervals (CIs) were reported. The variance inflation factor was calculated for all models to ensure the absence of multicollinearity. All analysis was performed using Python lifelines package version 27.8.

## Results

A total of 1007 patients completed the two-year follow-up per protocol, of whom 339 (33.7%) had a positive AI-ECG (predicting LVEF ≤ 40%) and 668 (66.3%) had a negative AI-ECG (predicting LVEF > 40%). A total of 84 recordings were true positives (TP), 653 recordings were true negatives (TN), 255 recordings were false positives (FP), and 15 recordings were false negatives (FN), as determined by comparing AI-ECG results with echocardiography findings. Overall, the mean age was 62.3 years (17.2), 528 (52.4%) patients were male, and 579 (57.5%) were White.

**Figure 1:**
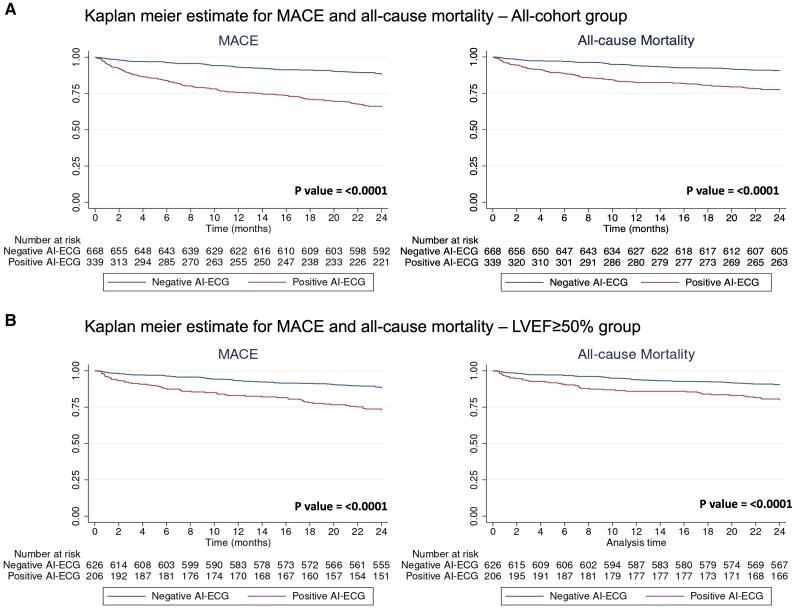
*P*-value was calculated via the Log-rank test. MACE, Major adverse cardiovascular events; AI-ECG, artificial intelligence-enhanced electrocardiogram.

As expected, comorbidities were more common in the group with an LVEF ≤ 40% (*Table 1*). Also as expected, for all patients, those with AI-ECG predicting LVEF ≤ 40% results had a higher rate of MACE than those with negative AI-ECG results (34.2% vs. 11.9%; *P* < 0.001; crude HR, 3.37 and 95% CI, 2.53–4.48; adjusted hazard ratio (aHR), 1.93 and 95% CI, 1.39–2.69; *Figure 1A*). These outcomes were driven by a higher rate of mortality (23% vs. 9.6%; *P* < 0.001; aHR, 1.56 and 95% CI, 1.06–2.29; *Figure 1B*) and HF hospitalization (14.5% vs. 1%; *P* < 0.001; *Table 2*).

**Table 1:** Baseline characteristics of study participants

	All participants (*n* = 1007)	LVEF ≤ 40 group (*n* = 99)	LVEF > 40 group (*n* = 908)	*P*-value
Age, years				
18–69	610 (60.5%)	47 (47.5%)	558 (61.5%)	0.009
≥70	397 (39.5%)	52 (52.5%)	350 (38.5%)	—
Mean (SD)	62.3 (17.2)	67.4 (14.9)	61.8 (17.4)	<0.001
Sex				
Male	528 (52.4%)	66 (66.7%)	462 (50.9%)	0.003
Female	479 (47.6%)	33 (33.3%)	446 (49.1%)	0.003
Ethnicity	—	—	—	0.295
Asian	200 (19.9%)	23 (23.2%)	177 (19.5%)	—
Black	93 (9.2%)	11 (11.1%)	82 (9%)	—
Mixed	21 (2%)	4 (4%)	17 (1.9%)	—
Other	114 (11.3%)	13 (13.1%)	101 (11.1%)	—
White	579 (57.5%)	48 (48.5%)	531 (58.5%)	—
Medical History				
Hypertension (HTN)	398 (39.5%)	55 (55.6%)	343 (37.8%)	<0.001
Myocardial Infarction	107 (10.6%)	38 (38.4%)	69 (7.6%)	<0.001
Atrial fibrillation^a^	169 (16.8%)	24 (24.2%)	145 (16%)	0.051
Ischaemic heart disease	114 (11.3%)	19 (19.2%)	95 (10.5%)	0.015
Pacemaker	42 (4.2%)	6 (6.1%)	36 (4%)	0.468
Diabetes	223 (22.1%)	39 (39.4%)	184 (20.3%)	<0.001
Stroke or transient ischaemic attack (TIA)	92 (9.1%)	14 (14.1%)	78 (8.6%)	0.102
Chronic kidney disease (CKD)	105 (10.4%)	23 (23.2%)	82 (9%)	<0.001
Smoking	145 (14.4%)	17 (17.2%)	128 (14.1%)	0.499
Excessive alcohol intake	28 (2.8%)	1 (1%)	27 (3%)	0.419
Hypercholesterolaemia	191 (19%)	30 (30.3%)	161 (17.7%)	<0.001
Pregnancy (current)	19 (1.9%)	0 (%)	19 (2.1%)	0.287
Valvular heart disease	386 (38.3%)	50 (50.5%)	336 (37%)	0.009
History of Heart Failure	96 (9.5%)	43 (43.4%)	53 (5.8%)	<0.001
HFrEF	48 (4.8%)	35 (35.3%)	13 (1.4%)	<0.001
HFmrEF	11 (1.1%)	5 (5.1%)	6 (0.6%)	<0.001
HFpEF	37 (3.7%)	3 (3%)	34 (3.7%)	0.719
Mean TTE LVEF (SD), %	54.6% (10.1)	29.9% (8.4)	57.3% (5.7)	<0.001
Indication of echocardiogram				
Suspicion of new heart failure (HF)	217	33 (33.3%)	184 (20.3%)	0.004
Re-evaluate known HF	65	32 (23.3%)	33 (3.6%)	<0.001
Hypertensive heart disease evaluation	15	2 (2%)	13 (1.4%)	0.982
Post-acute coronary syndrome	62	10 (10.1%)	52 (5.7%)	<0.001
Coronary artery disease	43	1 (1%)	42 (4.6%)	0.153
Arrhythmia	67	1 (1%)	66 (7.3%)	0.031
Valvular or structural heart disease (e.g. new murmur)	122	3 (3%)	119 (13.1%)	0.006
Valve assessment	51	6 (6.1%)	45 (5%)	0.814
Cardiac masses and thrombi	51	6 (6.1%)	45 (5%)	0.814
Congenital heart disease	1	0 (0%)	1 (0.1%)	—
Aortic stenosis	4	0 (0%)	4 (0.4%)	—
Syncope	28	1 (1%)	27 (3%)	0.419
Pericardial condition (e.g. effusion)	19	0 (0%)	19 (2.1%)	0.287
Stroke/TIA	47	1 (1%)	46 (5.1%)	0.117
Chemotherapy screening	47	1 (1%)	46 (5.1%)	0.117
Pre-operative screening	62	2 (2%)	60 (6.6%)	0.113
Pulmonary hypertension^b^	67	0 (0%)	67 (7.4%)	0.009
Other	38	0 (0%)	38 (4.2%)	0.072
Source of echocardiogram referral				<0.001
In-patient	364 (36.1%)	59 (59.6%)	305 (33.6%)	—
Out-patient	643 (63.9%)	40 (40.4%)	603 (66.4%)	—

Values are shown as numbers (%) unless otherwise stated. *P* values were calculated via Student’s t test or Pearson’s X^2^.

AI-ECG, Artificial Intelligence-enhanced electrocardiogram.

^a^Diagnoses of atrial fibrillation were identified based on the patients’ medical history, not the interpretation of the single-lead ECG.

^b^For patients who underwent echocardiography for pulmonary hypertension, the indication for echocardiography was a mixture of diagnostic and surveillance.

**Table 2:** Frequency table for all cohort

	Total	Positive AI-ECG (predicted LVEF ≤ 40%)	Negative AI-ECG (predicted LVEF > 40%)	*P*-value	
**All Cohort**	**(*n* = 1007)**	**(*n* = 339)**	**(*n* = 668)**	**(Log-rank test)**	**(Chi squared)**
MACE, *n* (%)	196 (19.5)	116 (34.2)	80 (11.9)	<0.001	<0.001
All-cause mortality, *n* (%)	142 (14.1)	78 (23)	64 (9.6)	<0.001	<0.001
HF hospitalization, *n* (%)	56 (5.6)	49 (14.5)	7 (1)	0.62	<0.001
Modified MACE, *n* (%)	181 (18.0)	111 (32.7)	70 (10.5)	<0.001	<0.001
**LVEF ≥ 50 Cohort**	**(*n* = 832)**	**(*n* = 206)**	**(*n* = 626)**	**(Log-rank test)**	**(Chi squared)**
MACE, *n* (%)	131 (15.7)	56 (27.2)	75 (11.9)	<0.001	<0.001
All-cause mortality, *n* (%)	102 (12.3)	42 (20.4)	60 (9.6)	<0.001	<0.001
HF hospitalization, *n* (%)	19 (2.3)	13 (6.3)	6 (0.9)	0.94	<0.001
Modified MACE, *n* (%)	118 (14.2)	53 (25.7)	65 (10.4)	<0.001	<0.001

Other components of MACE were reported in the appendix.

AI-ECG, Artificial intelligence-enhanced electrocardiogram; MACE, major adverse cardiovascular events; HF, Heart failure; LVEF, left ventricular ejection fraction.

Of the patients with LVEF ≥ 50% on echocardiography (*n* = 832), there was a higher MACE rate among those who were AI-ECG-positive (predicted LVEF ≤ 40%) (27.2% vs. 11.9%; *P* < 0.001; aHR, 1.71 and 95% CI, 1.18–2.47; *Figure 1B*). These results were largely influenced by a higher mortality rate (20.4% vs. 9.6%; *P* < 0.001; aHR, 1.59 and 95% CI, 1.05–2.42; *Figure 1B*). There was no significant difference between AI-ECG-predicted LVEF ≤ 40% and AI-ECG-predicted LVEF > 40% in the incidence of MI (2.1% vs. 0.3%; *P* = 0.17) and stroke or TIA (1.5% vs. 1.3%; *P*-value = 0.60). There was no significant difference in the incidence of MACE (Log-rank *P* = 0.12) or all-cause mortality (Log-rank *P* = 0.93) among the TP, TN, FP, and FN groups (determined based on conjoint use of AI-ECG and LVEF ≤ 40%)—Kaplan Meier estimate is detailed in Supplementary material online, Appendix  *Figure S2*.

In the univariable Cox regression analysis of all patients, the strongest predictor of MACE was AI-ECG-predicted LVEF ≤ 40% (crude HR, 3.37; 95% CI, 2.53–4.48) followed by CKD (crude HR, 3.23; 95% CI, 2.32–4.49), and history of HF (crude HR, 2.67; 95% CI, 1.96–3.89)—*Table 3*. Those predictors were consistent in the LVEF ≥ 50% group as well in predicting MACE (*Table 3*). Those predictors also show significant results in predicting all-cause mortality: CKD (crude HR, 4.05; 95% CI, 2.81–5.84), AI-ECG-predicted LVEF ≤ 40% (HR, 2.63; 95% CI, 1.89–3.66), history of HF (crude HR, 2.21; 95% CI, 1.45–3.35)—*Table 4*. A conjoint use of AI-ECG-predicted LVEF ≤ 40% and LVEF ≤ 40% (TP cases) showed a strong association with MACE (crude HR, 5.65; 95% CI, 3.86–8.28), all-cause mortality (crude HR, 3.04; 95% CI, 1.85–5.00), and modified MACE (crude HR, 5.71; 95% CI, 3.85–8.48)—Supplementary material online, Appendix  *Table S10*, *S11*, and *S16*.

**Table 3:** Cox regression model for MACE—all cohort and LVEF ≥ 50% group

Cox regression survival analysis	Univariable analysis		Multivariable analysis concordance index = 0.75	
	HR (95% CI)	*P*-value	HR (95% CI)	*P*-value
**All cohort (*n* = 1007)**				
Age (per 5-year increase)	1.22 (1.16–1.28)	<0.0001	1.03 (1.02–1.04)	<0.0001
Sex	1.27 (0.96–1.69)	0.0986	1.11 (0.83–1.48)	0.4992
LVEF% (per 5% decrease)	1.24 (1.18–1.30)	<0.001	1.12 (1.06–1.19)	0.0001
**Positive AI-ECG** **(predicting LVEF ≤ 40%)**	**3.37** (**2.53–4.48)**	**<0**.**001**	1.93 (1.39–2.69)	0.0001
Chronic kidney disease	3.23 (2.32–4.49)	<0.0001	1.91 (1.34–2.72)	0.0004
Cancer (active)	1.28 (0.85–1.90)	0.2335	1.42 (0.95–2.14)	0.0869
Hypercholesterolemia	0.94 (0.66–1.35)	0.7467	0.61 (0.42–0.88)	0.0084
Diabetes mellitus	2.24 (1.67–2.99)	<0.0001	1.50 (1.11–2.05)	0.0093
**LVEF ≥ 50% group (*n* = 832)**			**Concordance index = 0.74**	
Age (per 5-year increase)	1.23 (1.16–1.31)	<0.0001	1.03 (1.02–1.05)	<0.0001
Sex	1.35 (0.96–1.91)	0.0866	1.33 (0.93–1.89)	0.1171
**Positive AI-ECG** **(predicting LVEF ≤ 40%)**	2.52 (1.79–3.57)	<0.0001	1.71 (1.18–2.47)	0.0042
LVEF (per 5% decrease)	1.23 (1.01–1.50)	0.0445	1.14 (0.94–1.38)	0.1816
Valvular heart disease	2.05 (1.46–2.89)	<0.0001	1.53 (1.07–2.19)	0.0212
Diabetes mellitus	2.23 (1.55–3.21)	<0.0001	1.33 (0.93–1.89)	0.1171
Chronic kidney disease	2.87 (1.86–4.44)	<0.0001	1.93 (1.22–3.05)	0.0052
Cancer (active)	1.65 (1.07–2.55)	0.0248	1.78 (1.14–2.77)	0.0114
Hypercholesterolemia	1.01 (0.64–1.58)	0.9721	0.71 (0.44–1.14)	0.1536

**Table 4:** Cox regression model for all-cause mortality—all cohort and LVEF ≥ 50% group

Cox regression survival analysis	Univariable analysis		Multivariable analysis concordance index = 0.75	
	HR (95% CI)	*P*-value	HR (95% CI)	*P*-value
**All cohort (*n* = 1007)**				
Age (per 5-year increase)	1.22 (1.15–1.29)	<0.0001	1.03 (1.02–1.05)	<0.0001
Sex	1.35 (0.96–1.88)	0.0803	1.32 (0.94–1.87)	0.1091
LVEF (per 5% decrease)	1.18 (1.11–1.25)	<0.0001	1.11 (1.03–1.19)	0.0059
**Positive AI-ECG** **(predicting LVEF ≤ 40%)**	**2.63** (**1.89–3.66)**	**<0**.**0001**	1.56 (1.06–2.29)	0.0239
Atrial fibrillation	2.10 (1.46–3.03)	0.0001	1.34 (0.91–1.96)	0.1336
Valvular heart disease	1.76 (1.27–2.45)	0.0007	1.38 (0.97–1.94)	0.0704
Cardiac intervention	1.04 (0.69–1.58)	0.8375	0.57 (0.37–0.89)	0.0141
Chronic kidney disease	4.05 (2.81–5.84)	<0.0001	2.98 (2.02–4.38)	<0.0001
Cancer (active)	1.76 (1.15–2.69)	0.0093	1.93 (1.26–2.98)	0.0028
Stroke/Transient ischaemic attack	0.74 (0.39–1.42)	0.3683	0.56 (0.29–1.08)	0.0843
Hypercholesterolemia	0.80 (0.51–1.26)	0.3363	0.62 (0.39–0.98)	0.0423
**LVEF ≥ 50% group (*n* = 832)**			**Concordance index = 0.74**	
Age (per 5-year increase)	1.21 (1.13–1.30)	<0.0001	1.03 (1.02–1.05)	<0.0001
Sex	1.43 (0.96–2.11)	0.0764	1.48 (0.99–2.21)	0.0580
**Positive AI-ECG** **(predicting LVEF ≤ 40%)**	2.28 (1.54–3.38)	<0.0001	1.59 (1.05–2.42)	0.0300
LVEF (per 5% decrease)	1.24 (0.99–1.56)	0.0586	1.18 (0.95–1.48)	0.1433
Valvular heart disease	1.96 (1.33–2.89)	0.0007	1.62 (1.06–2.49)	0.0254
Diabetes mellitus	1.97 (1.30–2.99)	0.0015	1.42 (0.92–2.21)	0.1156
Chronic kidney disease	3.31 (2.06–5.30)	<0.0001	2.40 (1.45–3.98)	0.0007
Cancer (active)	2.05 (1.29–3.27)	0.0025	2.20 (1.37–3.54)	0.0011
Hypercholesterolemia	0.80 (0.46–1.39)	0.4347	0.61 (0.34–1.10)	0.0979
Cardiac intervention	0.96 (0.54–1.68)	0.8730	0.63 (0.34–1.14)	0.1236

In the multivariable Cox regression model, AI-ECG-predicted LVEF ≤ 40% was independently associated with higher MACE incidents (aHR, 1.93; 95% CI, 1.39–2.69—*Table 3*) and all-cause mortality (aHR, 1.56; 95% CI, 1.06–2.29—*Table 4*) in the overall group. For the group with echocardiographic LVEF ≥ 50%, AI-ECG-predicted LVEF ≤ 40% did show significant results for predicting MACE (aHR, 1.71; 95% CI, 1.18–2.47—*Table 3*), and all-cause mortality (aHR, 1.59; 95% CI, 1.05–2.42—*Table 4*). AI-ECG-predicted LVEF ≤ 40% demonstrated significant prognostic value for MACE and all-cause mortality, compared to echocardiographic LVEF, with higher HRs in both univariable and multivariable analyses (*Tables 3* and *4*). Significant observations of conjoint use of AI-ECG and LVEF 40 ≤% (TP cases) with MACE were observed (aHR, 3.96; 95% CI, 2.59–6.04), for all-cause mortality (aHR, 2.20; 95% CI, 1.24–3.90), and modified MACE (aHR, 3.83; 95% CI, 2.46–5.97)—Supplementary material online, Appendix  *Table S10*, *S11*, and *S16*. Additionally, AI-ECG-predicted LVEF ≤ 40% was independently associated with higher modified MACE (all-cause mortality or HF hospitalization) incidents in the overall group (aHR, 2.07; 95% CI, 1.47–2.92—*Table 5*), and in the LVEF ≥ 50% group (aHR, 1.82; 95% CI, 1.24–2.68—*Table 5*).

**Table 5:** Cox regression model for modified MACE—all cohort and LVEF ≥ 50% group

Cox regression survival analysis	Univariable analysis		Multivariable analysis concordance index = 0.76	
	HR (95% CI)	*P*-value	HR (95% CI)	*P*-value
**All cohort (*n* = 1007)**				
Age (per 5-year increase)	1.04 (1.03–1.05)	<0.0001	1.03 (1.02–1.04)	<0.0001
Sex	1.22 (0.91–1.64)	0.1788	1.09 (0.80–1.47)	0.5902
**Positive AI-ECG** **(predicting LVEF ≤ 40%)**	3.64 (2.70–4.91)	<0.0001	2.07 (1.47–2.92)	<0.0001
LVEF (per 5% decrease)	1.25 (1.19–1.31)	<0.0001	1.14 (1.07–1.21)	<0.0001
Diabetes mellitus	2.30 (1.70–3.11)	<0.0001	1.52 (1.11–2.10)	0.0098
Chronic kidney disease	3.59 (2.57–5.02)	<0.0001	2.04 (1.42–2.93)	0.0001
Cancer (active)	1.35 (0.90–2.03)	0.1496	1.54 (1.02–2.34)	0.0404
Cardiac intervention	1.47 (1.05–2.06)	0.0258	0.74 (0.51–1.06)	0.1044
Valvular heart disease	1.84 (1.38–2.47)	<0.0001	1.41 (1.03–1.92)	0.0299
Stroke/Transient ischaemic attack	0.89 (0.53–1.51)	0.6734	0.65 (0.38–1.11)	0.1165
Hypercholesterolemia	0.97 (0.67–1.40)	0.8566	0.67 (0.45–0.99)	0.0429
**LVEF ≥ 50% group (*n* = 832)**			**Concordance index = 0.75**	
Age (per 5-year increase)	1.04 (1.03–1.05)	<0.0001	1.03 (1.02–1.05)	<0.0001
Sex	1.32 (0.92–1.89)	0.1378	1.26 (0.87–1.83)	0.2153
**Positive AI-ECG** **(predicting LVEF ≤ 40%)**	2.74 (1.90–3.94)	<0.0001	1.82 (1.24–2.68)	0.0023
LVEF (per 5% decrease)	1.30 (1.05–1.60)	0.0170	1.22 (0.99–1.50)	0.0585
Diabetes mellitus	2.44 (1.67–3.55)	<0.0001	1.77 (1.18–2.65)	0.0058
Chronic kidney disease	3.30 (2.12–5.13)	<0.0001	2.19 (1.36–3.53)	0.0012
Cancer (active)	1.80 (1.15–2.81)	0.0104	1.98 (1.25–3.14)	0.0036
Cardiac intervention	1.18 (0.72–1.93)	0.5014	0.67 (0.40–1.12)	0.1279
Valvular heart disease	2.18 (1.52–3.14)	<0.0001	1.85 (1.25–2.72)	0.0019
Hypertension	1.41 (0.98–2.03)	0.0607	0.74 (0.49–1.11)	0.1493

AI-ECG probability score showed a significant association with MACE (HR, 1.31; 95% CI, 1.25–1.38) and all-cause mortality (HR, 1.25; 95% CI, 1.17–1.33) in the univariable cox regression analysis. Also, after adjusting with other variables, the AI-ECG probability score was significantly associated with MACE (HR, 1.17; 95% CI, 1.10–1.26), all-cause mortality (HR, 1.13; 95% CI, 1.04–1.22), and modified MACE (aHR, 1.17; 95% CI, 1.09–1.25—Supplementary material online, Appendix  *Table S14*). Other strong predictors are reported in Supplementary material online, Appendix Pages  *S8*–*S11*. AI-ECG results also extended to the LVEF ≥ 50% group showing a significant association with an increase in AI-ECG probability score. In the univariable Cox regression model, AI-ECG showed a significant result in predicting MACE (HR, 1.27; 95% CI, 1.18–1.36) and all-cause mortality (HR, 1.23; 95% CI, 1.13–1.34). In the multivariable Cox regression model, AI-ECG remained significant for predicting MACE (HR, 1.16; 95% CI, 1.07–1.26) and all-cause mortality (HR, 1.13, 95% CI, 1.03–1.24), and modified MACE (aHR, 1.17; 95% CI, 1.08–1.27—Supplementary material online, Appendix  *Table S15*). Also, AI-ECG demonstrated significant prognostic value for both MACE and all-cause mortality, while echocardiographic LVEF was not significantly associated with MACE or all-cause mortality after adjustment in this group (*Tables 3* and *4*). In our study, we systematically tested interaction terms between all variables included in the models, and none reached statistical significance (*P* > 0.05 for all). Additionally, we compared the performance of AI-ECG using ECG + PCG input (the current method within the manufacturer's specification; within the intended purpose approved by regulatory bodies including the Food and Drug Administration) against ECG input alone. The results showed no significant differences between the two methods (see Supplementary material online, Appendix  *Table S17*), indicating that the AI-ECG algorithm performance is not augmented by a PCG waveform input.

## Discussion

This study is the first to show that a single-lead AI-ECG designed to detect LVEF ≤ 40% identifies patients at risk of MACE and all-cause mortality independent of LVEF. We evaluated 1007 participants who had single-lead ECGs captured during routine echos and were followed up for two years. Unsurprisingly, an AI-ECG-predicted LVEF ≤ 40% screen was significantly associated with a higher incidence of MACE compared to an AI-ECG-predicted LVEF > 40% (34.2% vs. 11.9%; crude HR 3.37, 95% CI 2.53–4.48). However, when adjusting for potential covariates known to be associated with poorer patient outcomes including LVSD, the significant association between an AI-ECG-predicted LVEF ≤ 40% and a higher incidence of MACE persisted (aHR, 1.93; 95% CI, 1.39–2.69), indicating that this relationship is independent of LVEF results. Similarly, AI-ECG showed significant associations with all-cause mortality and modified MACE in both univariable and multivariable Cox regression analyses. This underscores the potential value of AI-ECG designed to detect LVEF 40≤% as an independent risk assessment tool for cardiovascular events during routine examinations.

Importantly, these findings extend to individuals with echocardiographic LVEF ≥ 50%. In patients with echocardiographic LVEF ≥ 50%, a positive AI-ECG (predictive of LVEF ≤ 40%) was an indicator of MACE, all-cause mortality, and modified MACE in the 2 years of follow-up. There was no clear link between AI-ECG and potential confounders (e.g. causes of death; indication of echo test; or source of referral) that could indicate a potential explanation for worse outcomes. As an illustration, patients undergoing echos as part of cancer screening were included, but no association was found to suggest an increased MACE and all-cause mortality risk associated with this subgroup.

The finding that a 10% increment in the AI-ECG probability score was independently associated with a 17% increase in the risk of MACE, and a 13% increase in the risk of all-cause mortality, indicates that features in the ECG that increase the likelihood of the AI algorithm detecting LVEF of ≤40% also correlate with a higher likelihood of detecting MACE and all-cause mortality. This would be expected but highlights the importance of not only presenting the binary AI-ECG results (positive or negative) but also reporting the probability scores, since these clearly provide additional prognostic value and can offer deeper insights into patient risk stratification. These findings also suggest important starting points for ongoing prospective studies using AI-ECG for longitudinal investigations in newly diagnosed patients with heart failure^10^ and highlight the importance of enhancing AI explainability to uncover new digital biomarkers linked to cardiovascular risk assessments. Building on emerging AI models that predict all-cause time of death from 12-lead ECG AI-ECG,^11^ AI-ECG when used in conjunction with an LVEF 40≤%, has also been shown to be associated with MACE but not with all-cause mortality. Further investigation is warranted to determine if AI-ECG can aid in risk assessment among patients with different LVEF classifications or severe LV impairment, or those who may progress to this, positioning AI-ECG as an ideal community screening tool for prioritizing urgent follow-up in routine echo tests.^12^

This study has several clinical implications. First, enhanced AI-ECG prediction tools are now accessible through single-lead ECG technology (such as is currently in wide-scale implementation in routine clinical care in the NHS, NCT05987670). These tools provide an opportunity for enhanced risk stratification, particularly in community or resource-limited settings where access to multi-lead ECGs or echocardiography may be limited. By identifying patients at high risk of MACE or all-cause mortality, single-lead AI-ECG can help guide further diagnostic workup, such as prioritizing echocardiography or specialist referrals. This highlights the potential of AI-ECG as a complementary tool for risk stratification. Second, with the increasing consumer/wearable technologies preferring single-lead ECG capability, this may ultimately empower patients to play an active role in managing their health and may lead to better early detection of cardiac issues, improved chronic disease management, and incremental patient satisfaction and confidence.^13^ Third, the probability score highlights the meaningful insights that can be gleaned from AI-ECG models beyond their intrinsic purpose. These scores can help stratify patients across a continuum of risk, potentially enabling personalized follow-up strategies. Fourth, our findings underscore the potential for AI-ECG to complement existing diagnostic tools rather than replace them. In cases where an echo has already been performed, single-lead ECG offers an additional dimension of risk stratification. AI-ECG captures subtle morphological and temporal changes in the electrical activity of the heart that may not directly correlate with traditional echocardiographic parameters. AI-ECG has also demonstrated significant prognostic value even in patients with preserved LVEF, identifying risks that echocardiographic LVEF alone may not capture. Notably, the higher HRs observed with AI-ECG compared to echocardiographic LVEF suggest a stronger association with adverse outcomes. However, it is important to note that this comparison was limited to LVEF—a single echocardiographic measure commonly used in clinical practice. While our findings suggest that AI-ECG can detect risks that may not be fully captured by echocardiographic LVEF, i.e. in patients with preserved LVEF, other echocardiographic parameters, such as chamber dimensions, and valvular function, also contribute to risk stratification and associated with adverse outcomes.^14–16^ This complementary role highlights the value of AI-ECG as a cost-effective, scalable screening tool that could streamline the allocation of healthcare resources by identifying patients who would benefit most from further resting or intervention. The obvious evolution of this work is towards an AI algorithm specifically trained for identifying MACE and all-cause mortality using a single-lead ECG, which would naturally be expected to have superior performance to what we have derived from the AI-ECG algorithm trained for LVEF ≤ 40%.

AI-ECG's association with a higher incidence of MACE and all-cause mortality would naturally be expected given the strong correlation with a reduced ejection fraction of 40% or less, a known predictor of cardiovascular death.^17,18^ The capacity to identify MACE in LVEF > 50% likely relates to disease—cardiovascular (e.g. ischaemic or rhythm abnormalities) or otherwise—that affects the ECG morphology and has impacted the AI-ECG results. For all-cause mortality, this likely reflects the AI-ECG output being influenced by ECG patterns that may signify early myocardial stress, electrical remodelling, or latent cardiac dysfunction. This observation aligns with findings from other studies that have attempted to describe the ECG features that most inform AI-ECG model outputs.^19–22^ Using interpretability methods such as saliency mapping and gradient-weighted class activation mapping, these studies have demonstrated that AI-ECG models often index heavily on features such as QRS complex morphology or T-wave characteristics, to predict cardiovascular outcomes. Importantly, these relationships are not linear; instead, they represent complex, multidimensional interactions that extend beyond conventional clinical interpretation. This suggests that much more subtle morphological features—beyond the human brain’s capability to detect—are at play. Furthermore, other studies have suggested that AI-ECG can potentially identify subtle cardiac dysfunction that is not readily observable on echocardiography, since AI-ECG has been shown to be associated with a four-fold higher risk of developing ventricular dysfunction in previous studies.^1,7^ Ultimately, the potential clinical benefits of this technology may outweigh concerns around a lack of explainability, though methods for this are continuing to evolve.^23,24^

Our study has limitations. First, although we had comprehensive data including comorbidities, not all potential confounders, such as various disease severity and additional echocardiographic parameters, were accounted for. Second, we have limited false negative results (negative AI-ECG and impaired LVEF; *n* = 15) which prohibits us from performing survival analysis for including risk assessment for sub-analyses investigation of AI-ECG with conjoint use of LVEF ≤ 40%. Third, some of the adjusted predictors lack sufficient statistical power due to the small proportion of patients within certain subgroups and the heterogeneous nature of these groups (e.g. history of HF). Additionally, while patients with pacemakers (*n* = 42) were included in the study, the small sample size precluded a detailed analysis of the AI algorithm’s performance on pacemaker rhythms, which requires further investigation in a larger cohort. Also, variation in the sample size of AI-ECG probability score (e.g. each 10% variation) is limited and not normally distributed and therefore limits further investigation. Therefore, a larger prospective study is needed to evaluate AI-ECG with the conjoint use of LVSD (LVEF ≤ 40%) and AI-ECG probability score as a risk stratification tool. Also, the intra- and inter-patient variability of the exact recording position (vector) is a limitation shared by repeat 12-lead ECGs, though perhaps to a lesser degree when using an ECG-enabled stethoscope since only two fixed electrodes are being applied. Lastly, despite evidence of AI-ECG’s ability to identify individuals at risk of MACE, a notable limitation is the constrained explainability of the AI-ECG algorithm, which could impede its widespread clinical adoption, especially in complex clinical decision-making processes that directly affect patient care.

## Conclusion

In conclusion, our study found that AI-ECG designed to detect LVEF ≤ 40% can identify patients at risk of MACE and all-cause mortality from single-lead ECG in the absence of LVSD. Such capabilities may allow further risk stratification for patients undergoing clinical assessment. Further prospective investigations with a larger sample size are needed to validate the AI-ECG probability score and its conjoint use with LVEF% to identify patients at risk of MACE and mortality.

## Supplementary Material

ztaf057_Supplementary_Data

## Data Availability

Study data may be shared for future research collaborations upon request. Interested researchers should contact the corresponding author. Data access will be granted in accordance with the data protection policies of Imperial College London and Imperial College Healthcare NHS Trust.
