# Supplementary material for: Artificial intelligence analysis of the single-lead ECG predicts long-term clinical outcomes
Source: Eur Heart J Digit Health. 2025 Jun 9;6(4):635–44. doi: 10.1093/ehjdh/ztaf057 (PMC12282343; doi:10.1093/ehjdh/ztaf057)
Supplement: ztaf057_Supplementary_Data [file ztaf057_supplementary_data.docx]

**Supplementary appendix**

**Contents:**

| **Supplementary item** | **Page** |
| --- | --- |
| AI-ECG output | 2 |
| Kaplan meier estimate for MACE and all-cause mortality for conjoint use of AI-ECG and LVEF ≤ 40% – All-cohort group | 3 |
| Frequency table for all-cohort | 4 |
| Heatmap correlation | 5 |
| Cox regression model for MACE – all cohort | 6 |
| Cox regression model for all-cause mortality – all cohort | 7 |
| Cox regression model for MACE – LVEF ≥50% group | 8 |
| Cox regression model for all-cause mortality – LVEF ≥50% group | 9 |
| Cox regression model for MACE – all cohort (AI-ECG probability score) | 10 |
| Cox regression model for all-cause mortality – all cohort (AI-ECG probability score) | 11 |
| Cox regression model for MACE – LVEF ≥50% group (AI-ECG probability score) | 12 |
| Cox regression model for all-cause mortality – LVEF ≥50% group (AI-ECG probability score) | 13 |
| Cox regression model for MACE – all cohort (AI-ECG conjoint use with LVEF ≤40%) | 14 |
| Cox regression model for all-cause mortality – all cohort (AI-ECG conjoint use with LVEF ≤40%) | 15 |
| Cox regression model for modified MACE – all cohort | 16 |
| Cox regression model for modified MACE – LVEF ≥50% group | 17 |
| Cox regression model for modified MACE – all cohort (AI-ECG probability score) | 18 |
| Cox regression model for modified MACE – LVEF ≥50% group (AI-ECG probability score) | 19 |
| Cox regression model for modified MACE – all cohort (AI-ECG conjoint use with LVEF ≤40%) | 20 |
| Comparison of AI-ECG performance using ECG + PCG vs. ECG only for MACE, all-cause Mortality, and modified MACE in all cohort and LVEF ≥50% group | 21 |


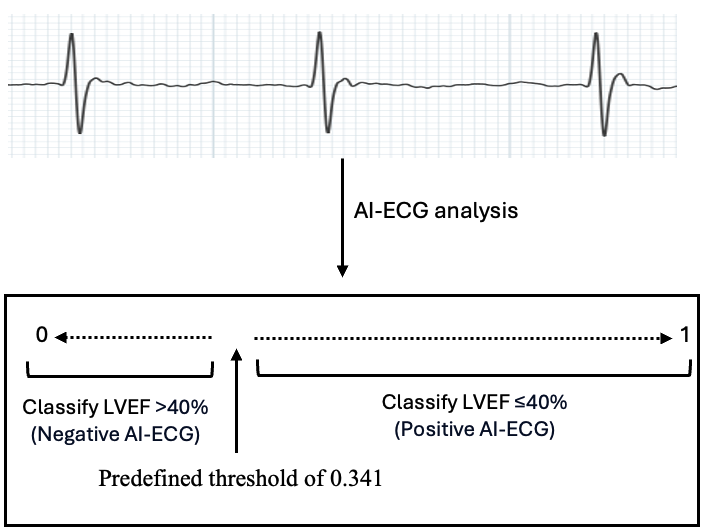
Figure 1. AI-ECG output

**AI-ECG:** Artificial intelligence-enhanced electrocardiogram; **CNN**: Convolutional neural network; **LVEF**: Left ventricular ejection fraction. A 0.341 threshold established from a previous study.


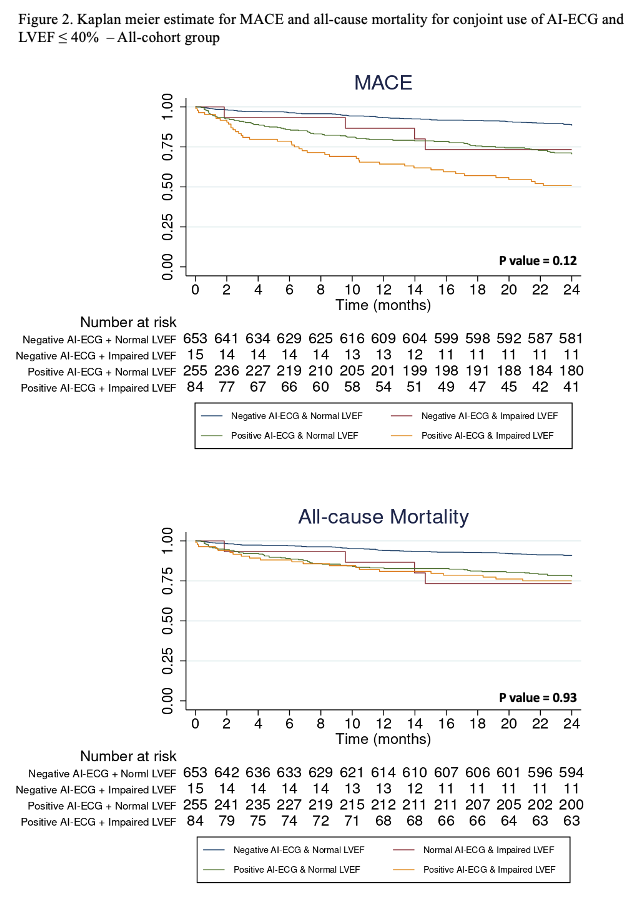


P value was calculated via Log rank test. **MACE:** Major Adverse Cardiovascular Events; **AI-ECG:** Artificial Intelligence-enhanced electrocardiogram; **LVEF:** Left Ventricular Ejection Fraction.

|  | **Total** | **Positive AI-ECG** | **Negative AI-ECG** | **P value** | **P value** |
| --- | --- | --- | --- | --- | --- |
| **All Cohort** | **(n = 1007)** | **(n = 339)** | **(n = 668)** | **(Log rank test)** | **(Chi squared)** |
| Acute MI, n (%) | 9 (0.9) | 7 (2.1) | 2 (0.3) | 0.17 | 0.014 |
| Stroke or TIA, n (%) | 14 (1.4) | 5 (1.5) | 9 (1.3) | 0.60 | 1.000 |
| **LVEF ≥50 Cohort** | **(n = 832)** | **(n = 206)** | **(n = 626)** | **(Log rank test)** | **(Chi squared)** |
| Acute MI, n (%) | 4 (0.5) | 2 (0.9) | 2 (0.3) | 0.43 | 0.554 |
| Stroke or TIA, n (%) | 13 (1.6) | 4 (1.9) | 9 (1.4) | 0.54 | 0.855 |

Table 1 Frequency table for all-cohort


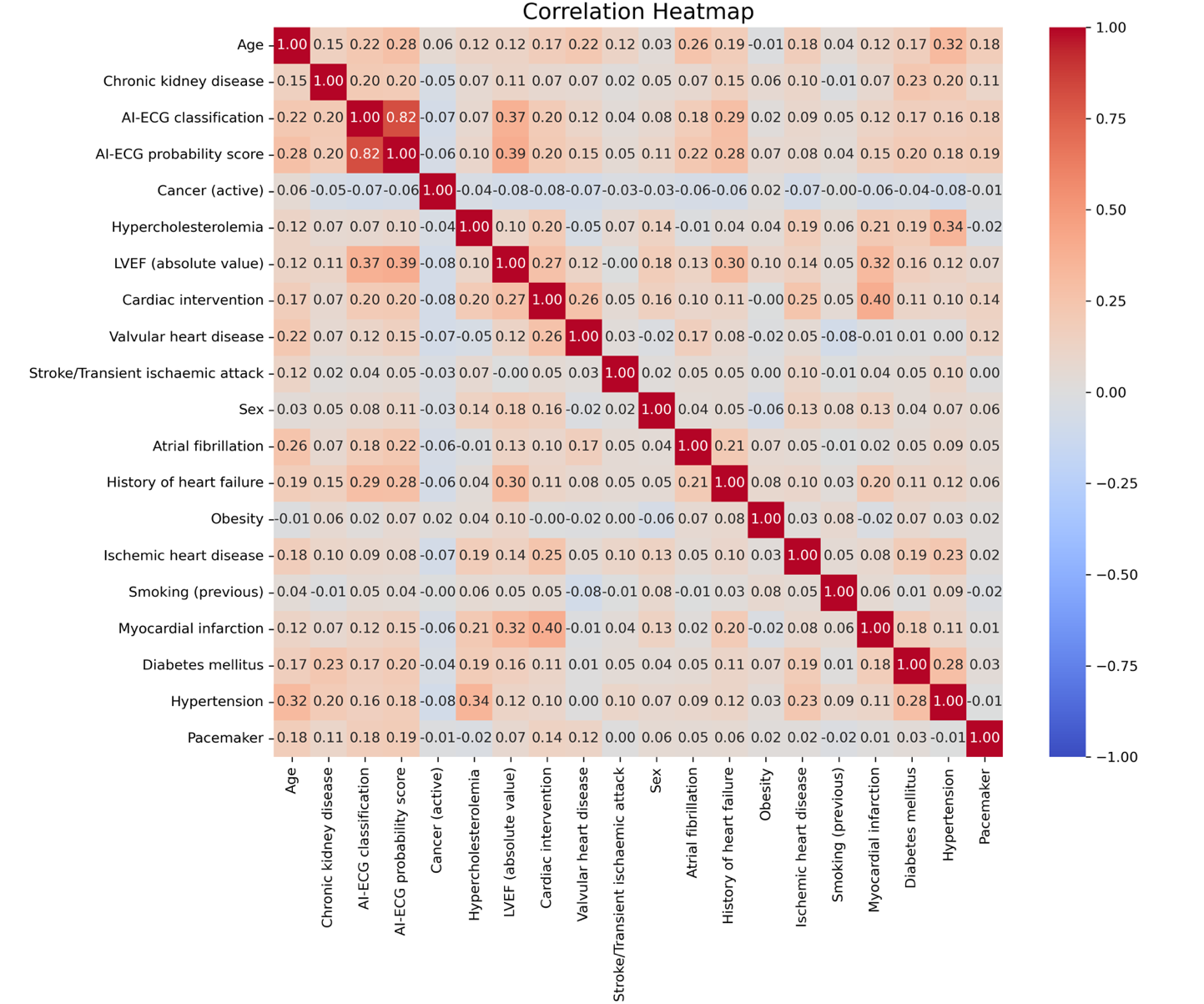


Figure 3 Correlation Heatmap of Clinical variables

The heatmap illustrates the pairwise Pearson/Spearman correlation coefficients between various clinical variables included in the study. The colour gradient represents the strength and direction of the correlations, with red indicating positive correlations and blue indicating negative correlations. **AI-ECG:** Artificial Intelligence-enhanced electrocardiogram; **LVEF:** Left ventricular ejection fraction.

| Cox regression survival analysis | Univariable analysis | | Multivariable analysis concordance index = 0.75 | |
| --- | --- | --- | --- | --- |
|  | HR (95% CI) | P value | HR (95% CI) | P value |
| Age (per 5-year increase) | 1.22 (1.16 – 1.28) | <0.0001 | 1.03 (1.02 - 1.04) | <0.0001 |
| Sex | 1.27 (0.96 – 1.69) | 0.0986 | 1.11 (0.83 - 1.48) | 0.4992 |
| **Positive AI-ECG**  **(predicting LVEF ≤40%)** | **3.37 (2.53 – 4.48)** | **<0.001** | 1.93 (1.39 - 2.69) | 0.0001 |
| LVEF% (per 5% decrease) | 1.24 (1.18 - 1.30) | <0.0001 | 1.12 (1.06 - 1.19) | 0.0001 |
| Chronic kidney disease | 3.23 (2.32 – 4.49) | <0.0001 | 1.91 (1.34 - 2.72) | 0.0004 |
| Cancer (active) | 1.28 (0.85 – 1.90) | 0.2335 | 1.42 (0.95 - 2.14) | 0.0869 |
| Hypercholesterolemia | 0.94 (0.66 – 1.35) | 0.7467 | 0.61 (0.42 - 0.88) | 0.0084 |
| Diabetes mellitus | 2.24 (1.67 – 2.99) | <0.0001 | 1.50 (1.11 - 2.05) | 0.0093 |
| Valvular heart disease | 1.32 (1.32 – 2.31) | 0.0001 |  |  |
| Hypertension | 1.72 (1.30 – 2.27) | 0.0002 |  |  |
| Cardiac intervention | 1.44 (1.04 – 2.00) | 0.0268 |  |  |
| Pacemaker | 1.99 (1.16 – 3.43) | 0.0131 |  |  |
| History of heart failure | 2.76 (1.96 – 3.89) | <0.0001 |  |  |
| Obesity | 1.41 (0.82 – 2.43) | 0.2136 |  |  |
| Stroke/Transient ischaemic attack | 1.00 (0.62 – 1.63) | 0.9891 |  |  |
| Ischemic heart disease | 1.33 (0.89 – 1.99) | 0.1606 |  |  |
| Myocardial infarction | 1.75 (1.20 – 2.56) | 0.0036 |  |  |
| Smoking previous | 1.27 (0.82 – 1.98) | 0.2903 |  |  |
| Atrial fibrillation | 1.99 (1.45 – 2.73) | <0.0001 |  |  |

Table 2 Cox regression model for MACE – all cohort

Table 3 Cox regression model for all-cause mortality – all cohort

| Cox regression survival analysis | Univariable analysis | | Multivariable analysis concordance index = 0.75 | |
| --- | --- | --- | --- | --- |
|  | HR (95% CI) | P value | HR (95% CI) | P value |
| Age (per 5-year increase) | 1.22 (1.15 – 1.29) | <0.0001 | 1.03 (1.02 - 1.05) | <0.0001 |
| Sex | 1.35 (0.96 – 1.88) | 0.0803 | 1.32 (0.94 - 1.87) | 0.1091 |
| LVEF (per 5% decrease) | 1.18 (1.11 - 1.25) | <0.0001 | 1.11 (1.03 - 1.19) | 0.0059 |
| **Positive AI-ECG**  **(predicting LVEF ≤40%)** | **2.63 (1.89 – 3.66)** | **<0.0001** | 1.56 (1.06 - 2.29) | 0.0239 |
| Atrial fibrillation | 2.10 (1.46 – 3.03) | 0.0001 | 1.34 (0.91 - 1.96) | 0.1336 |
| Valvular heart disease | 1.76 (1.27 – 2.45) | 0.0007 | 1.38 (0.97 - 1.94) | 0.0704 |
| Cardiac intervention | 1.04 (0.69 – 1.58) | 0.8375 | 0.57 (0.37 - 0.89) | 0.0141 |
| Chronic kidney disease | 4.05 (2.81 – 5.84) | <0.0001 | 2.98 (2.02 - 4.38) | <0.0001 |
| Cancer (active) | 1.76 (1.15 – 2.69) | 0.0093 | 1.93 (1.26 - 2.98) | 0.0028 |
| Stroke/Transient ischaemic attack | 0.74 (0.39 – 1.42) | 0.3683 | 0.56 (0.29 - 1.08) | 0.0843 |
| Hypercholesterolemia | 0.80 (0.51 – 1.26) | 0.3363 | 0.62 (0.39 - 0.98) | 0.0423 |
| Hypertension | 1.45 (1.04 – 2.01) | 0.0283 |  |  |
| Diabetes mellitus | 1.82 (1.29 – 2.59) | 0.0007 |  |  |
| Obesity | 0.90 (0.42 – 1.93) | 0.7902 |  |  |
| Ischemic heart disease | 0.93 (0.54 – 1.58) | 0.7760 |  |  |
| Smoking previous | 1.17 (0.68 – 1.99) | 0.5745 |  |  |
| Myocardial infarction | 1.24 (0.76 – 2.03) | 0.3954 |  |  |
| History of heart failure | 2.21 (1.45 – 3.35) | 0.0002 |  |  |
| Pacemaker | 2.08 (1.12 – 3.85) | 0.0197 |  |  |

Table 4 Cox regression model for MACE – LVEF ≥50% group

| Cox regression survival analysis | Univariable analysis | | Multivariable analysis concordance index = 0.74 | |
| --- | --- | --- | --- | --- |
|  | HR (95% CI) | P value | HR (95% CI) | P value |
| Age (per 5-year increase) | 1.23 (1.16 – 1.31) | <0.0001 | 1.03 (1.02 - 1.05) | <0.0001 |
| Sex | 1.35 (0.96 – 1.91) | 0.0866 | 1.33 (0.93 - 1.89) | 0.1171 |
| **Positive AI-ECG**  **(predicting LVEF ≤40%)** | 2.52 (1.79 – 3.57) | <0.0001 | 1.71 (1.18 - 2.47) | 0.0042 |
| LVEF (per 5% decrease) | 1.23 (1.01 - 1.50) | 0.0445 | 1.14 (0.94 - 1.38) | 0.1816 |
| Valvular heart disease | 2.05 (1.46 – 2.89) | <0.0001 | 1.53 (1.07 - 2.19) | 0.0212 |
| Diabetes mellitus | 2.23 (1.55 – 3.21) | <0.0001 | 1.33 (0.93 - 1.89) | 0.1171 |
| Chronic kidney disease | 2.87 (1.86 – 4.44) | <0.0001 | 1.93 (1.22 - 3.05) | 0.0052 |
| Cancer (active) | 1.65 (1.07 – 2.55) | 0.0248 | 1.78 (1.14 - 2.77) | 0.0114 |
| Hypercholesterolemia | 1.01 (0.64 – 1.58) | 0.9721 | 0.71 (0.44 - 1.14) | 0.1536 |
| Hypertension | 1.48 (1.05 – 2.09) | 0.0255 |  |  |
| Cardiac intervention | 1.25 (0.79 – 1.97) | 0.3427 |  |  |
| Obesity | 1.49 (0.78 – 2.85) | 0.2220 |  |  |
| Stroke/Transient ischaemic attack | 1.09 (0.60 – 1.97) | 0.7865 |  |  |
| Ischemic heart disease | 1.26 (0.75 – 2.13) | 0.3830 |  |  |
| Smoking previous | 0.97 (0.52 – 1.80) | 0.9241 |  |  |
| Myocardial infarction | 0.82 (0.36 – 1.85) | 0.6298 |  |  |
| Atrial fibrillation | 2.00 (1.34 – 2.98) | 0.0007 |  |  |
| Pacemaker | 2.23 (1.13 – 4.39) | 0.0202 |  |  |
| History of heart failure | 2.38 (1.34 – 4.21) | 0.0031 |  |  |

Table 5 Cox regression model for all-cause mortality – LVEF ≥50% group

| Cox regression survival analysis | Univariable analysis | | Multivariable analysis concordance index = 0.74 | |
| --- | --- | --- | --- | --- |
|  | HR (95% CI) | P value | HR (95% CI) | P value |
| Age (per 5-year increase) | 1.21 (1.13 – 1.30) | <0.0001 | 1.03 (1.02 - 1.05) | <0.0001 |
| Sex | 1.43 (0.96 – 2.11) | 0.0764 | 1.48 (0.99 - 2.21) | 0.0580 |
| **Positive AI-ECG**  **(predicting LVEF ≤40%)** | 2.28 (1.54 – 3.38) | <0.0001 | 1.59 (1.05 - 2.42) | 0.0300 |
| LVEF (per 5% decrease) | 1.24 (0.99 - 1.56) | 0.0586 | 1.18 (0.95 - 1.48) | 0.1433 |
| Valvular heart disease | 1.96 (1.33 – 2.89) | 0.0007 | 1.62 (1.06 - 2.49) | 0.0254 |
| Diabetes mellitus | 1.97 (1.30 – 2.99) | 0.0015 | 1.42 (0.92 - 2.21) | 0.1156 |
| Chronic kidney disease | 3.31 (2.06 – 5.30) | <0.0001 | 2.40 (1.45 - 3.98) | 0.0007 |
| Cancer (active) | 2.05 (1.29 – 3.27) | 0.0025 | 2.20 (1.37 - 3.54) | 0.0011 |
| Hypercholesterolemia | 0.80 (0.46 – 1.39) | 0.4347 | 0.61 (0.34 - 1.10) | 0.0979 |
| Cardiac intervention | 0.96 (0.54 – 1.68) | 0.8730 | 0.63 (0.34 - 1.14) | 0.1236 |
| Pacemaker | 2.12 (0.99 – 4.58) | 0.0542 |  |  |
| Atrial fibrillation | 1.96 (1.25 – 3.07) | <0.0001 |  |  |
| Obesity | 1.10 (0.48 – 2.52) | 0.8135 |  |  |
| Stroke/Transient ischaemic attack | 0.79 (0.36 – 1.69) | 0.5390 |  |  |
| Ischemic heart disease | 1.09 (0.58 – 2.03) | 0.7975 |  |  |
| Smoking previous | 0.88 (0.43 – 1.82) | 0.7357 |  |  |
| Hypertension | 1.25 (0.85 – 1.86) | 0.2604 |  |  |
| Myocardial infarction | 0.70 (0.26 – 1.89) | 0.4783 |  |  |
| History of heart failure | 2.55 (1.37 – 4.78) | <0.0001 |  |  |

Table 6 Cox regression model for MACE – all cohort (AI-ECG probability score)

| Cox regression survival analysis | Univariable analysis | | Multivariable analysis concordance index = 0.75 | |
| --- | --- | --- | --- | --- |
|  | HR (95% CI) | P value | HR (95% CI) | P value |
| Age (per 5-year increase) | 1.22 (1.16 – 1.28) | <0.0001 | 1.03 (1.02 - 1.04) | <0.0001 |
| Sex | 1.27 (0.96 – 1.69) | 0.0986 | 1.10 (0.83 - 1.47) | 0.5096 |
| **AI-ECG (per 10% increase)** | **1.31 (1.25 – 1.38)** | **<0.0001** | 1.17 (1.10 - 1.26) | <0.0001 |
| LVEF (per 5% decrease) | 1.24 (1.18 - 1.30) | <0.0001 | 1.08 (1.02 - 1.15) | 0.0136 |
| Diabetes mellitus | 2.24 (1.67 – 2.99) | <0.0001 | 1.50 (1.11 - 2.04) | 0.0090 |
| Chronic kidney disease | 3.23 (2.32 – 4.49) | <0.0001 | 1.84 (1.29 - 2.63) | 0.0007 |
| Cancer (active) | 1.28 (0.85 – 1.90) | 0.2335 | 1.37 (0.92 - 2.05) | 0.1262 |
| Hypercholesterolemia | 0.94 (0.66 – 1.35) | 0.7467 | 0.62 (0.43 - 0.90) | 0.0120 |
| Stroke/Transient ischaemic attack | 1.00 (0.62 – 1.63) | 0.9891 | 0.71 (0.44 - 1.16) | 0.1718 |
| Valvular heart disease | 1.75 (1.32 – 2.31) | 0.0001 |  |  |
| Pacemaker | 1.99 (1.16 – 3.43) | 0.0131 |  |  |
| Cardiac intervention | 1.44 (1.04 – 2.00) | 0.0268 |  |  |
| Atrial fibrillation | 1.99 (1.45 – 2.73) | <0.0001 |  |  |
| Hypertension | 1.72 (1.30 – 2.27) | 0.0002 |  |  |
| Myocardial infarction | 1.75 (1.20 – 2.56) | 0.0036 |  |  |
| Smoking previous | 1.27 (0.82 – 1.98) | 0.2903 |  |  |
| History of heart failure | 2.76 (1.96 – 3.89) | <0.0001 |  |  |
| Ischemic heart disease | 1.33 (0.89 – 1.99) | 0.1606 |  |  |
| Obesity | 1.41 (0.82 – 2.43) | 0.2136 |  |  |

Table 7 Cox regression model for all-cause mortality – all cohort (AI-ECG probability score)

| Cox regression survival analysis | Univariable analysis | | Multivariable analysis concordance index = 0.74 | |
| --- | --- | --- | --- | --- |
|  | HR (95% CI) | P value | HR (95% CI) | P value |
| Age (per 5-year increase) | 1.22 (1.15 – 1.29) | <0.0001 | 1.03 (1.02 - 1.05) | <0.0001 |
| Sex | 1.35 (0.96 – 1.88) | 0.0803 | 1.32 (0.94 - 1.86) | 0.1127 |
| **AI-ECG (per 10% increase)** | **1.25 (1.17 – 1.33)** | **<0.0001** | 1.13 (1.04 - 1.22) | 0.0026 |
| LVEF (per 5% decrease) | 1.18 (1.11 - 1.25) | <0.0001 | 1.07 (0.99 - 1.16) | 0.0867 |
| Valvular heart disease | 1.76 (1.27 – 2.45) | 0.0007 | 1.35 (0.96 - 1.91) | 0.0889 |
| Chronic kidney disease | 4.05 (2.81 – 5.84) | <0.0001 | 2.95 (2.00 - 4.34) | <0.0001 |
| Cancer (active) | 1.76 (1.15 – 2.69) | 0.0093 | 1.87 (1.22 - 2.88) | 0.0042 |
| Hypercholesterolemia | 0.80 (0.51 – 1.26) | 0.3363 | 0.60 (0.37 - 0.95) | 0.0290 |
| Cardiac intervention | 1.04 (0.69 – 1.58) | 0.8375 | 0.58 (0.37 - 0.90) | 0.0152 |
| Stroke/Transient ischaemic attack | 0.74 (0.39 – 1.42) | 0.3683 | 0.56 (0.29 - 1.08) | 0.0853 |
| Atrial fibrillation | 2.10 (1.46 – 3.03) | 0.0001 |  |  |
| Pacemaker | 2.08 (1.12 – 3.85) | 0.0197 |  |  |
| Hypertension | 1.45 (1.04 – 2.01) | 0.0283 |  |  |
| History of heart failure | 2.21 (1.45 – 3.35) | 0.0002 |  |  |
| Obesity | 0.90 (0.42 – 1.93) | 0.7902 |  |  |
| Ischemic heart disease | 0.93 (0.54 – 1.58) | 0.7760 |  |  |
| Smoking previous | 1.17 (0.68 – 1.99) | 0.5745 |  |  |
| Diabetes mellitus | 1.82 (1.29 – 2.59) | 0.0007 |  |  |
| Myocardial infarction | 1.24 (0.76 – 2.03) | 0.3948 |  |  |

Table 8 Cox regression model for MACE – LVEF ≥50% group (AI-ECG probability score)

| Cox regression survival analysis | Univariable analysis | | Multivariable analysis concordance index = 0.74 | |
| --- | --- | --- | --- | --- |
|  | HR (95% CI) | P value | HR (95% CI) | P value |
| Age (per 5-year increase) | 1.23 (1.16 – 1.31) | <0.0001 | 1.03 (1.02 - 1.05) | <0.0001 |
| Sex | 1.35 (0.96 – 1.91) | 0.0866 | 1.31 (0.92 - 1.88) | 0.1319 |
| **AI-ECG (per 10% increase)** | **1.27 (1.18 – 1.36)** | **<0.0001** | 1.16 (1.07 - 1.25) | 0.0003 |
| LVEF (per 5% decrease) | 1.23 (1.01 - 1.50) | 0.0445 | 1.12 (0.93 - 1.36) | 0.2395 |
| Valvular heart disease | 2.05 (1.46 – 2.89) | <0.0001 | 1.49 (1.04 - 2.14) | 0.0305 |
| Diabetes mellitus | 2.23 (1.55 – 3.21) | <0.0001 | 1.62 (1.11 - 2.36) | 0.0123 |
| Chronic kidney disease | 2.87 (1.86 – 4.44) | <0.0001 | 1.85 (1.17 - 2.93) | 0.0090 |
| Cancer (active) | 1.65 (1.07 – 2.55) | 0.0248 | 1.80 (1.15 - 2.80) | 0.0096 |
| Hypercholesterolemia | 1.01 (0.64 – 1.58) | 0.9721 | 0.69 (0.43 - 1.12) | 0.1329 |
| Atrial fibrillation | 2.00 (1.34 – 2.98) | 0.0007 |  |  |
| Pacemaker | 2.23 (1.13 – 4.39) | 0.0202 |  |  |
| Hypertension | 1.48 (1.05 – 2.09) | 0.0255 |  |  |
| Cardiac intervention | 1.25 (0.79 – 1.97) | 0.3427 |  |  |
| History of heart failure | 2.38 (1.34 – 4.21) | 0.0031 |  |  |
| Obesity | 1.49 (0.78 – 2.85) | 0.2220 |  |  |
| Stroke/Transient ischaemic attack | 1.09 (0.60 – 1.97) | 0.7865 |  |  |
| Ischemic heart disease | 1.26 (0.75 – 2.13) | 0.3830 |  |  |
| Smoking previous | 0.97 (0.52 – 1.80) | 0.9241 |  |  |
| Myocardial infarction | 0.82 (0.36 – 1.85) | 0.6289 |  |  |

Table 9 Cox regression model for all-cause mortality – LVEF ≥50% group (AI-ECG probability score)

| Cox regression survival analysis | Univariable analysis | | Multivariable analysis concordance index = 0.73 | |
| --- | --- | --- | --- | --- |
|  | HR (95% CI) | P value | HR (95% CI) | P value |
| Age (per 5-year increase) | 1.21 (1.13 – 1.30) | <0.0001 | 1.03 (1.02 - 1.05) | <0.0001 |
| Sex | 1.43 (0.96 – 2.11) | 0.0764 | 1.48 (0.99 - 2.21) | 0.0592 |
| **AI-ECG (per 10% increase)** | **1.23 (1.13 – 1.34)** | **<0.0001** | 1.13 (1.03 - 1.24) | 0.0073 |
| LVEF (per 5% decrease) | 1.24 (0.99 - 1.56) | 0.0586 | 1.17 (0.93 - 1.46) | 0.1747 |
| Valvular heart disease | 1.96 (1.33 – 2.89) | 0.0007 | 1.58 (1.03 - 2.42) | 0.0358 |
| Diabetes mellitus | 1.97 (1.30 – 2.99) | 0.0015 | 1.46 (0.94 - 2.26) | 0.0925 |
| Chronic kidney disease | 3.31 (2.06 – 5.30) | <0.0001 | 2.31 (1.39 - 3.84) | 0.0013 |
| Cancer (active) | 2.05 (1.29 – 3.27) | 0.0025 | 2.21 (1.38 - 3.56) | 0.0010 |
| Hypercholesterolemia | 0.80 (0.46 – 1.39) | 0.4347 | 0.60 (0.33 - 1.08) | 0.0881 |
| Cardiac intervention | 0.96 (0.54 – 1.68) | 0.8730 | 0.62 (0.34 - 1.12) | 0.1123 |
| Atrial fibrillation | 1.96 (1.25 – 3.07) | <0.0001 |  |  |
| Pacemaker | 2.12 (0.99 – 4.58) | 0.0542 |  |  |
| History of heart failure | 2.55 (1.37 – 4.78) | <0.0001 |  |  |
| Obesity | 1.10 (0.48 – 2.52) | 0.8135 |  |  |
| Stroke/Transient ischaemic attack | 0.79 (0.36 – 1.69) | 0.5390 |  |  |
| Ischemic heart disease | 1.09 (0.58 – 2.03) | 0.7975 |  |  |
| Smoking previous | 0.88 (0.43 – 1.82) | 0.7357 |  |  |
| Hypertension | 1.25 (0.85 – 1.86) | 0.2604 |  |  |
| Myocardial infarction | 0.70 (0.26 – 1.89) | 0.4783 |  |  |

Table 10 Cox regression model for MACE – all cohort (AI-ECG conjoint use with LVEF 40≤%)

| Cox regression survival analysis | Univariable analysis | | Multivariable analysis concordance index = 0.73 | |
| --- | --- | --- | --- | --- |
|  | HR (95% CI) | P value | HR (95% CI) | P value |
| Age (per 5-year increase) | 1.20 (1.13 – 1.28) | <0.0001 | 1.03 (1.02 - 1.04) | <0.0001 |
| Sex | 1.59 (1.09 – 2.30) | 0.0152 | 1.40 (0.95 - 2.06) | 0.0863 |
| **Positive AI-ECG & LVEF ≤40%** | **5.65 (3.86 – 8.28)** | **<0.0001** | 3.96 (2.59 - 6.04) | <0.0001 |
| Diabetes mellitus | 2.74 (1.89 – 3.99) | <0.0001 | 1.94 (1.30 - 2.89) | 0.0011 |
| Chronic kidney disease | 3.10 (1.95 – 4.93) | <0.0001 | 1.60 (0.96 - 2.64) | 0.0689 |
| Cancer (active) | 1.58 (0.99 – 2.51) | 0.0547 | 1.52 (0.94 - 2.45) | 0.0852 |
| Hypercholesterolemia | 1.03 (0.64 – 1.63) | 0.9151 | 0.61 (0.38 - 0.98) | 0.0425 |
| Ischemic heart disease | 1.18 (0.69 – 2.03) | 0.5502 | 0.55 (0.31 - 0.98) | 0.0424 |
| Hypertension | 1.59 (1.11 – 2.29) | 0.0122 |  |  |
| Cardiac intervention | 1.55 (1.01 – 2.39) | 0.0446 |  |  |
| History of heart failure | 2.76 (1.75 – 4.42) | <0.0001 |  |  |
| Myocardial infarction | 2.01 (1.23 – 3.29) | 0.0053 |  |  |
| Obesity | 1.15 (0.53 – 2.46) | 0.7265 |  |  |
| Stroke/Transient ischaemic attack | 1.09 (0.60 – 1.98) | 0.7833 |  |  |
| Valvular heart disease | 1.51 (1.05 – 2.17) | 0.0274 |  |  |
| Smoking previous | 1.27 (0.71 – 2.26) | 0.4169 |  |  |
| Atrial fibrillation | 1.59 (1.00 – 2.52) | 0.0515 |  |  |
| Pacemaker | 1.62 (0.60 – 4.40) | 0.3412 |  |  |

Table 11 Cox regression model for all-cause mortality – all cohort (AI-ECG conjoint use with LVEF ≤40%)

| Cox regression survival analysis | Univariable analysis | | Multivariable analysis concordance index = 0.73 | |
| --- | --- | --- | --- | --- |
|  | HR (95% CI) | P value | HR (95% CI) | P value |
| Age (per 5-year increase) | 1.18 (1.10 – 1.27) | <0.0001 | 1.03 (1.02 - 1.05) | 0.0002 |
| Sex | 1.88 (1.19 – 2.97) | 0.0068 | 1.92 (1.20 - 3.07) | 0.0066 |
| **Positive AI-ECG + LVEF ≤40%** | **3.04 (1.85 – 5.00)** | **<0.0001** | 2.20 (1.24 - 3.90) | 0.0069 |
| Diabetes mellitus | 2.27 (1.43 – 3.58) | 0.0005 | 1.62 (0.99 - 2.65) | 0.0564 |
| Chronic kidney disease | 4.32 (2.61 – 7.16) | <0.0001 | 3.16 (1.79 - 5.56) | 0.0001 |
| Cancer (active) | 2.43 (1.48 – 3.99) | 0.0005 | 2.28 (1.37 - 3.81) | 0.0016 |
| Ischemic heart disease | 0.51 (0.21 – 1.26) | 0.1468 | 0.33 (0.13 - 0.85) | 0.0217 |
| Cardiac intervention | 1.03 (0.58 – 1.84) | 0.9144 | 0.54 (0.28 - 1.03) | 0.0604 |
| Stroke/Transient ischaemic attack | 0.61 (0.25 – 1.52) | 0.2896 | 0.40 (0.16 - 1.00) | 0.0497 |
| Atrial fibrillation | 1.67 (0.96 – 2.88) | 0.0670 |  |  |
| Pacemaker | 2.35 (0.86 – 6.42) | 0.0957 |  |  |
| Hypertension | 1.27 (0.81 – 1.97) | 0.2956 |  |  |
| Valvular heart disease | 1.42 (0.92 – 2.20) | 0.1156 |  |  |
| Obesity | 0.65 (0.21 – 2.07) | 0.4669 |  |  |
| Smoking previous | 1.43 (0.74 – 2.77) | 0.2927 |  |  |
| Hypercholesterolemia | 1.01 (0.58 – 1.77) | 0.9662 |  |  |
| Myocardial infarction | 1.36 (0.70 – 2.63) | 0.3673 |  |  |
| History of heart failure | 1.53 (0.79 – 2.97) | 0.2073 |  |  |

Table 12 Cox regression model for modified MACE – all cohort

| Cox regression survival analysis | Univariable analysis | | Multivariable analysis concordance index = 0.76 | |
| --- | --- | --- | --- | --- |
|  | HR (95% CI) | P value | HR (95% CI) | P value |
| Age (per 5-year increase) | 1.04 (1.03 - 1.05) | <0.0001 | 1.03 (1.02 - 1.04) | <0.0001 |
| Sex | 1.22 (0.91 - 1.64) | 0.1788 | 1.09 (0.80 - 1.47) | 0.5902 |
| **Positive AI-ECG**  **(predicting LVEF ≤40%)** | 3.64 (2.70 - 4.91) | <0.0001 | 2.07 (1.47 - 2.92) | <0.0001 |
| LVEF (per 5% decrease) | 1.25 (1.19 - 1.31) | <0.0001 | 1.14 (1.07 - 1.21) | <0.0001 |
| Diabetes mellitus | 2.30 (1.70 - 3.11) | <0.0001 | 1.52 (1.11 - 2.10) | 0.0098 |
| Chronic kidney disease | 3.59 (2.57 - 5.02) | <0.0001 | 2.04 (1.42 - 2.93) | 0.0001 |
| Cancer (active) | 1.35 (0.90 - 2.03) | 0.1496 | 1.54 (1.02 - 2.34) | 0.0404 |
| Cardiac intervention | 1.47 (1.05 - 2.06) | 0.0258 | 0.74 (0.51 - 1.06) | 0.1044 |
| Valvular heart disease | 1.84 (1.38 - 2.47) | <0.0001 | 1.41 (1.03 - 1.92) | 0.0299 |
| Stroke/Transient ischaemic attack | 0.89 (0.53 - 1.51) | 0.6734 | 0.65 (0.38 - 1.11) | 0.1165 |
| Hypercholesterolemia | 0.97 (0.67 - 1.40) | 0.8566 | 0.67 (0.45 - 0.99) | 0.0429 |
| Atrial fibrillation | 2.14 (1.55 - 2.96) | <0.0001 |  |  |
| Pacemaker | 2.18 (1.27 - 3.77) | 0.0050 |  |  |
| Hypertension | 1.65 (1.23 - 2.21) | 0.0008 |  |  |
| Obesity | 1.42 (0.81 - 2.49) | 0.2270 |  |  |
| Smoking previous | 1.25 (0.79 - 1.99) | 0.3438 |  |  |
| Myocardial infarction | 1.78 (1.20 - 2.63) | 0.0040 |  |  |
| History of heart failure | 2.95 (2.08 - 4.18) | <0.0001 |  |  |
| Ischemic heart disease | 1.41 (0.94 - 2.13) | 0.0983 |  |  |

Table 13 Cox regression model for modified MACE – LVEF ≥50% group

| Cox regression survival analysis | Univariable analysis | | Multivariable analysis concordance index = 0.75 | |
| --- | --- | --- | --- | --- |
|  | HR (95% CI) | P value | HR (95% CI) | P value |
| Age (per 5-year increase) | 1.04 (1.03 - 1.05) | <0.0001 | 1.03 (1.02 - 1.05) | <0.0001 |
| Sex | 1.32 (0.92 - 1.89) | 0.1378 | 1.26 (0.87 - 1.83) | 0.2153 |
| **Positive AI-ECG**  **(predicting LVEF ≤40%)** | 2.74 (1.90 - 3.94) | <0.0001 | 1.82 (1.24 - 2.68) | 0.0023 |
| LVEF (per 5% decrease) | 1.30 (1.05 - 1.60) | 0.0170 | 1.22 (0.99 - 1.50) | 0.0585 |
| Diabetes mellitus | 2.44 (1.67 - 3.55) | <0.0001 | 1.77 (1.18 - 2.65) | 0.0058 |
| Chronic kidney disease | 3.30 (2.12 - 5.13) | <0.0001 | 2.19 (1.36 - 3.53) | 0.0012 |
| Cancer (active) | 1.80 (1.15 - 2.81) | 0.0104 | 1.98 (1.25 - 3.14) | 0.0036 |
| Cardiac intervention | 1.18 (0.72 - 1.93) | 0.5014 | 0.67 (0.40 - 1.12) | 0.1279 |
| Valvular heart disease | 2.18 (1.52 - 3.14) | <0.0001 | 1.85 (1.25 - 2.72) | 0.0019 |
| Hypertension | 1.41 (0.98 - 2.03) | 0.0607 | 0.74 (0.49 - 1.11) | 0.1493 |
| Stroke/Transient ischaemic attack | 0.88 (0.45 - 1.74) | 0.7123 |  |  |
| Hypercholesterolemia | 1.02 (0.64 - 1.64) | 0.9231 |  |  |
| Atrial fibrillation | 2.20 (1.46 - 3.31) | 0.0002 |  |  |
| Pacemaker | 2.51 (1.27 - 4.96) | 0.0079 |  |  |
| Obesity | 1.49 (0.75 - 2.94) | 0.2508 |  |  |
| Smoking previous | 0.98 (0.51 - 1.88) | 0.9564 |  |  |
| Myocardial infarction | 0.75 (0.31 - 1.85) | 0.5378 |  |  |
| History of heart failure | 2.69 (1.51 - 4.78) | 0.0008 |  |  |
| Ischemic heart disease | 1.33 (0.77 - 2.29) | 0.3015 |  |  |

Table 14 Cox regression model modified MACE – all cohort (AI-ECG probability score)

| Cox regression survival analysis | Univariable analysis | | Multivariable analysis concordance index = 0.76 | |
| --- | --- | --- | --- | --- |
|  | HR (95% CI) | P value | HR (95% CI) | P value |
| Age (per 5-year increase) | 1.04 (1.03 - 1.05) | <0.0001 | 1.03 (1.02 - 1.04) | <0.0001 |
| Sex | 1.22 (0.91 - 1.64) | 0.1788 | 1.04 (0.77 - 1.41) | 0.7792 |
| **AI-ECG (per 10% increase)** | 1.32 (1.25 - 1.40) | <0.0001 | 1.17 (1.09 - 1.25) | <0.0001 |
| LVEF (per 5% decrease) | 1.25 (1.19 - 1.31) | <0.0001 | 1.09 (1.02 - 1.17) | 0.0078 |
| Diabetes mellitus | 2.30 (1.70 - 3.11) | <0.0001 | 1.49 (1.08 - 2.05) | 0.0139 |
| Chronic kidney disease | 3.59 (2.57 - 5.02) | <0.0001 | 2.04 (1.42 - 2.92) | 0.0001 |
| Cancer (active) | 1.35 (0.90 - 2.03) | 0.1496 | 1.48 (0.98 - 2.25) | 0.0618 |
| Atrial fibrillation | 2.14 (1.55 - 2.96) | <0.0001 | 1.31 (0.93 - 1.83) | 0.1194 |
| Hypercholesterolemia | 0.97 (0.67 - 1.40) | 0.8566 | 0.65 (0.45 - 0.96) | 0.0302 |
| Stroke/Transient ischaemic attack | 0.89 (0.53 - 1.51) | 0.6734 | 0.64 (0.37 - 1.09) | 0.0982 |
| Cardiac intervention | 1.47 (1.05 - 2.06) | 0.0258 |  |  |
| Valvular heart disease | 1.84 (1.38 - 2.47) | <0.0001 |  |  |
| History of heart failure | 2.95 (2.08 - 4.18) | <0.0001 |  |  |
| Obesity | 1.42 (0.81 - 2.49) | 0.2270 |  |  |
| Ischemic heart disease | 1.41 (0.94 - 2.13) | 0.0983 |  |  |
| Smoking previous | 1.25 (0.79 - 1.99) | 0.3438 |  |  |
| Myocardial infarction | 1.78 (1.20 - 2.63) | 0.0040 |  |  |
| Hypertension | 1.65 (1.23 - 2.21) | 0.0008 |  |  |
| Pacemaker | 2.18 (1.27 - 3.77) | 0.0050 |  |  |

Table 15 Cox regression model for modified MACE – LVEF ≥50% group (AI-ECG probability score)

| Cox regression survival analysis | Univariable analysis | | Multivariable analysis concordance index = 0.76 | |
| --- | --- | --- | --- | --- |
|  | HR (95% CI) | P value | HR (95% CI) | P value |
| Age (per 5-year increase) | 1.04 (1.03 - 1.05) | <0.0001 | 1.03 (1.02 - 1.05) | <0.0001 |
| Sex | 1.32 (0.92 - 1.89) | 0.1378 | 1.25 (0.87 - 1.81) | 0.2335 |
| **AI-ECG (per 10% increase)** | 1.28 (1.19 - 1.38) | <0.0001 | 1.17 (1.08 - 1.27) | 0.0002 |
| LVEF (per 5% decrease) | 1.30 (1.05 - 1.60) | 0.0170 | 1.20 (0.98 - 1.48) | 0.0799 |
| Diabetes mellitus | 2.44 (1.67 - 3.55) | <0.0001 | 1.84 (1.23 - 2.75) | 0.0032 |
| Chronic kidney disease | 3.30 (2.12 - 5.13) | <0.0001 | 2.10 (1.30 - 3.38) | 0.0025 |
| Cancer (active) | 1.80 (1.15 - 2.81) | 0.0104 | 2.01 (1.27 - 3.18) | 0.0030 |
| Cardiac intervention | 1.18 (0.72 - 1.93) | 0.5014 | 0.66 (0.39 - 1.10) | 0.1118 |
| Valvular heart disease | 2.18 (1.52 - 3.14) | <0.0001 | 1.81 (1.23 - 2.66) | 0.0027 |
| Hypertension | 1.41 (0.98 - 2.03) | 0.0607 | 0.73 (0.49 - 1.10) | 0.1342 |
| Stroke/Transient ischaemic attack | 0.88 (0.45 - 1.74) | 0.7123 |  |  |
| Hypercholesterolemia | 1.02 (0.64 - 1.64) | 0.9231 |  |  |
| Atrial fibrillation | 2.20 (1.46 - 3.31) | 0.0002 |  |  |
| Pacemaker | 2.51 (1.27 - 4.96) | 0.0079 |  |  |
| Obesity | 1.49 (0.75 - 2.94) | 0.2508 |  |  |
| Smoking previous | 0.98 (0.51 - 1.88) | 0.9564 |  |  |
| Myocardial infarction | 0.75 (0.31 - 1.85) | 0.5378 |  |  |
| History of heart failure | 2.69 (1.51 - 4.78) | 0.0008 |  |  |
| Ischemic heart disease | 1.33 (0.77 - 2.29) | 0.3015 |  |  |

Table 16 Cox regression model for modified MACE – all cohort (AI-ECG conjoint use with LVEF ≤40%)

| Cox regression survival analysis | Univariable analysis | | Multivariable analysis concordance index = 0.79 | |
| --- | --- | --- | --- | --- |
|  | HR (95% CI) | P value | HR (95% CI) | P value |
| Age (per 5-year increase) | 1.03 (1.02 - 1.05) | <0.0001 | 1.03 (1.01 - 1.04) | 0.0003 |
| Sex | 1.45 (0.98 - 2.12) | 0.0606 | 1.23 (0.83 - 1.83) | 0.3107 |
| **Positive AI-ECG & LVEF ≤40%** | 5.71 (3.85 - 8.48) | <0.0001 | 3.83 (2.46 - 5.97) | <0.0001 |
| Chronic kidney disease | 3.52 (2.20 - 5.63) | <0.0001 | 1.78 (1.06 - 2.97) | 0.0286 |
| Diabetes mellitus | 3.25 (2.21 - 4.78) | <0.0001 | 2.16 (1.42 - 3.27) | 0.0003 |
| Cancer (active) | 1.67 (1.03 - 2.69) | 0.0359 | 1.68 (1.03 - 2.75) | 0.0395 |
| Hypercholesterolemia | 1.14 (0.72 - 1.83) | 0.5764 | 0.69 (0.42 - 1.11) | 0.1283 |
| Ischemic heart disease | 1.34 (0.78 - 2.32) | 0.2873 | 0.66 (0.37 - 1.19) | 0.1665 |
| Stroke/Transient ischaemic attack | 0.95 (0.49 - 1.82) | 0.8734 | 0.62 (0.32 - 1.20) | 0.1573 |
| Cardiac intervention | 1.65 (1.06 - 2.56) | 0.0265 |  |  |
| Atrial fibrillation | 1.85 (1.16 - 2.96) | 0.0102 |  |  |
| History of heart failure | 2.93 (1.82 - 4.73) | <0.0001 |  |  |
| Obesity | 1.30 (0.61 - 2.80) | 0.4986 |  |  |
| Valvular heart disease | 1.59 (1.09 - 2.32) | 0.0168 |  |  |
| Smoking previous | 1.17 (0.63 - 2.19) | 0.6127 |  |  |
| Myocardial infarction | 2.21 (1.35 - 3.64) | 0.0017 |  |  |
| Hypertension | 1.49 (1.02 - 2.18) | 0.0399 |  |  |
| Pacemaker | 1.88 (0.69 - 5.10) | 0.2159 |  |  |

Table 17. Comparison of AI-ECG performance using ECG + PCG vs. ECG only for MACE, all-cause Mortality, and modified MACE in all cohort and LVEF ≥50% group

| **AI-ECG output** | **MACE**  **(all cohort; n = 809*)** | **MACE**  **(LVEF ≥ 50%; n = 683*)** |
| --- | --- | --- |
| **ECG + PCG (aHR**, 95% CI)** | 2.02 (1.41 – 2.91) | 1.81 (1.21 – 2.70) |
| **ECG Only (aHR**, 95% CI)** | 1.79 (1.26 – 2.55) | 1.63 (1.11 – 2.42) |
| **Z-test (HR comparison)** | Z = 0.464, p = 0.6428 | Z = 0.350, p = 0.7264 |
| **C-index (ECG + PCG)** | 0.7583 | 0.7571 |
| **C-index (ECG Only)** | 0.7482 | 0.7516 |
| **Z-test (C-index)** | Z = 0.710, p = 0.4778 | Z = 0.389, p = 0.6974 |
|  | **All-cause mortality**  **(all cohort; n = 809*)** | **All-cause mortality**  **(LVEF ≥ 50%; n = 683*)** |
| **ECG + PCG (aHR**, 95% CI)** | 1.60 (1.04 – 2.45) | 1.52 (0.96 – 2.41) **^†^** |
| **ECG Only (aHR**, 95% CI)** | 1.64 (1.08 – 2.49) | 1.48 (0.94 – 2.32) **^†^** |
| **Z-test (HR comparison)** | Z = -0.084, p = 0.9331 | Z = 0.075, p = 0.9400 |
| **C-index (ECG + PCG)** | 0.7631 | 0.7631 |
| **C-index (ECG Only)** | 0.7628 | 0.7632 |
| **Z-test (C-index)** | Z = 0.025, p = 0.9803 | Z = -0.003, p = 0.9974 |
|  | **Modified MACE**  **(all cohort; n = 809*)** | **Modified MACE**  **(LVEF ≥ 50%; n = 683*)** |
| **ECG + PCG (aHR**, 95% CI)** | 2.00 (1.35 – 2.96) | 1.87 (1.22 – 2.87) |
| **ECG Only (aHR**, 95% CI)** | 1.67 (1.14 – 2.44) | 1.54 (1.01 – 2.33) |
| **Z-test (HR comparison)** | Z = 0.659, p = 0.5097 | Z = 0.651, p = 0.5151 |
| **C-index (ECG + PCG)** | 0.7720 | 0.7674 |
| **C-index (ECG Only)** | 0.7641 | 0.7588 |
| **Z-test (C-index)** | Z = 0.565, p = 0.5719 | Z = 0.608, p = 0.5430 |

**(*):** All data with poor-quality PCG were excluded from the analyses

**(**):** All the reported Cox regression models were adjusted to the same covariates.

(**†):** P value was not significant
